# A Public Health Scourge: The Recent Surge of an Ancient Disease

**DOI:** 10.1155/crip/5799790

**Published:** 2026-04-15

**Authors:** Abigail Pleiss, Mikale Kuntz, Selly Strauch, Susan Roe

**Affiliations:** ^1^ Department of Pathology, School of Medicine and Health Sciences, University of North Dakota, Grand Forks, North Dakota, USA, und.edu

**Keywords:** congenital syphilis, placentomegaly, public health crisis, stillborn infant

## Abstract

The recent resurgence of congenital syphilis is striking, with an increase in cases 10‐fold over the past 12 years, including a 30% rise within the past 3 years alone. The consequences of untreated maternal syphilis can be severe, contributing to significant fetal and neonatal illness and death in cases of congenital syphilis. We present a case involving a male fetus delivered stillborn at 33 weeks′ gestation to a 30‐year‐old woman with limited prenatal care. The mother had been diagnosed with late latent syphilis 8 days before delivery and had a history of polysubstance use. Ultrasound at 32 weeks and 5 days revealed intrauterine fetal demise. Fetal measurements corresponded to approximately 27 weeks′ gestation and demonstrated significant hydrops. Postmortem examination showed maceration, enlarged placenta, and focal necrotizing funisitis. Additional findings included arteritis of chorionic plate vessels and placental changes consistent with maternal vascular malperfusion. Radiographic evaluation identified lucent metaphyseal bands in the long bones. Immunohistochemical staining detected spirochetal organisms within the lung and placenta tissues. This case highlights the profound clinical implications of congenital syphilis and reinforces the necessity of consistent prenatal screening and prompt treatment of maternal infection.

## 1. Introduction

Syphilis prevalence reached its peak just prior to the widespread introduction of penicillin in 1947. However, recent trends have shown exponential increases. In 2022, there was an increase in congenital syphilis cases by nearly one‐third from the previous year alone, totaling 3761 reported cases. This upward trend coincided with a rise in primary and secondary syphilis rates among females ages 15–44, which climbed from 16.3 to 19.1 cases per 100,000 [1]. In the context of a more than tenfold escalation in congenital syphilis incidence in the United States over the past decade, and the persistent decline of other perinatal infections such as human immunodeficiency virus and hepatitis B in the United States, these trends reflect a substantial and ongoing breakdown in syphilis prevention, screening, and treatment efforts that fail to protect pregnant patients and their infants [1–4].

*Treponema pallidum*, a spirochete and causative agent of the systemic infection syphilis, is transmitted sexually or vertically during pregnancy. Early recognition of syphilis can be challenging as its presentation can be variable and sometimes understated [4–6]. Children born to women with untreated syphilis are at risk of congenital syphilis, acquired in utero [6]. Congenital syphilis can have devastating impacts on neonatal morbidity and mortality. This report will discuss a case of congenital syphilis, address the clinical impact of congenital syphilis, and highlight the importance of adequate prenatal screening and treatment.

## 2. Case Report

We present a case of a male fetus delivered stillborn to a 30‐year‐old G6P1323 mother, self‐identified as American Indian/Alaska Native. The pregnancy had been complicated by limited prenatal care. The prenatal course was complicated by late latent syphilis, diagnosed only 8 days prior to delivery, and polysubstance use disorder including methamphetamine and heroin. The mother was receiving Suboxone and residing in a rehabilitation program at the time of the presentation. Intrauterine fetal demise was diagnosed at 32 weeks and 5 days by ultrasound, with biometry consistent with a 27‐week fetus with significant hydrops. Initial maternal laboratory results included elevated AST 87 U/L, ALT 78 U/L, and TSH 5.3 mIU/L. She had no prior history of hypothyroidism and was started on thyroid replacement postpartum. Unfortunately, it is unknown whether or not the patient′s mother was able to complete treatment and if the partner completed testing as well.

**Figure 1:**
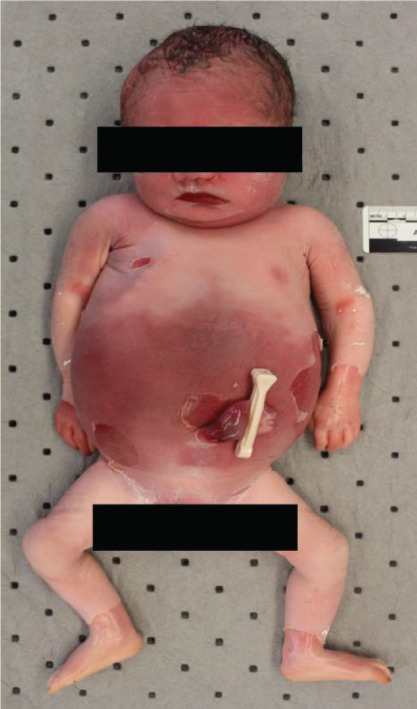
Maceration with skin slippage and discoloration.

**Figure 2:**
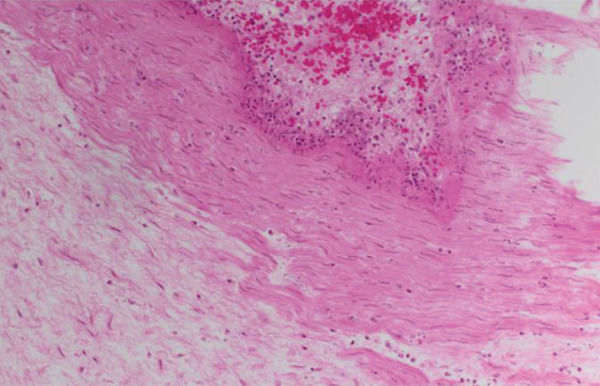
Focal necrotizing funisitis seen in umbilical artery.

**Figure 3:**
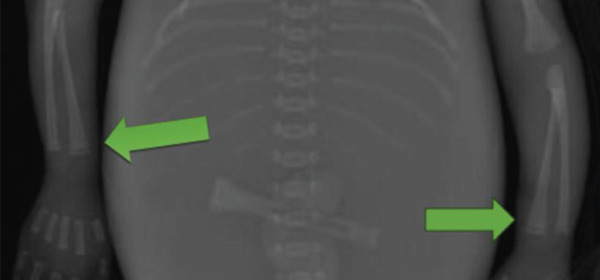
Radiographic lucent bands of the long bones.

Autopsy of the stillborn infant was performed, and significant findings included maceration with skin slippage and discoloration, features of prematurity, and findings of presumed congenital syphilis (Figure 1). There was evidence of hydrops fetalis, placentomegaly, mild choriodeciduitis, acute arteritis in chorionic plate vessels, and an edematous umbilical cord with focal necrotizing funisitis (Figure 2). Radiographic trophic lucent bands of the long bones were seen (Figure 3).

Upon further examination of the placenta, there were reported features of relatively severe maternal vascular malperfusion, specifically accelerated maturation and paucity of villi, and increased syncytial knots. There was acute arteritis of the larger chorionic plate vessels and villous stromal karyorrhexis. Chronic stimulant abuse (polysubstance use and chronic vasoconstriction) and the risk of stillbirth, as well as small‐for‐gestational‐age fetuses and neonates [5], are notable noninfectious conditions associated with these histopathological placental findings.

Specimens were forwarded to the Minnesota Department of Health for syphilis testing, who agreed with the diagnostic features of congenital infection by *T. pallidum*. Immunohistochemical testing by the CDC confirmed spirochete organisms in the lungs and placenta [20].

## 3. Discussion

Congenital syphilis occurs from the vertical transmission of *T. pallidum* from mother to fetus during pregnancy or delivery, which may occur through transplacental spread or direct contact with infectious maternal lesions [5–7]. Although transmission can happen at any stage of pregnancy, the risk increases with increasing gestation [5, 7]. Untreated primary and secondary syphilis are associated with the highest risk of transmission, whereas fetal infection occurs less frequently during latent stages of disease [5]. These differences are thought to reflect both organism burden and the duration of fetal exposure [7].

### 3.1. Clinical Manifestations

Maternal syphilis infection during pregnancy has been linked to a wide range of adverse outcomes, including preterm delivery, fetal growth restriction, and stillbirth. Neonatal outcomes include intrauterine growth restriction, small for gestational age, and nonimmune hydrops fetalis. Certain abnormalities suggestive of infection in utero may be seen on prenatal fetal ultrasound. These findings include placentomegaly, polyhydramnios, fetal hydrops, and abnormal Doppler studies, which have all been described in association with congenital syphilis and may prompt further evaluation [8]. Serological syphilis testing for neonates and their mothers is also suggested for infants delivered with placentas that are markedly enlarged relative to their birth weight, exceeding the 90th percentile, as a majority of these cases are found to have congenital syphilis [9, 10].

When cases result in stillbirth, the autopsy serves to establish the final diagnosis. The most common autopsy findings include hydrops fetalis, enlargement of the liver and spleen, abnormal thymus development, and metaphyseal abnormalities. Placental examination may reveal increased placental weight, inflammatory changes involving the membranes and decidua, and villous abnormalities [8]. The presence of enlarged and hypercellular villi, proliferative vascular changes, and either acute or chronic villitis constitute the “syphilis triad” [11]. Autopsy findings can be relatively nonspecific, and definitive diagnosis of congenital syphilis ultimately depends on the identification of *T. pallidum* using silver stain or immunohistochemistry [12].

In cases of atypical manifestations of congenital syphilis, there may be missed diagnoses. Furthermore, insurance often does not cover autopsy in the context of stillbirth, which may contribute to additional missed cases if an autopsy is not performed [13]. In cases of live birth, findings may not be readily apparent as most neonates do not show outward signs of infection at delivery. The two syndromes of clinical congenital disease vary with the timing of disease expression and can be divided into early congenital syphilis and late congenital syphilis [7, 14]. Children with early congenital syphilis develop symptoms within the first 2 years of life. In addition to the perinatal outcomes listed above, manifestations include but are not limited to failure to thrive, hepatomegaly, splenomegaly, jaundice, anemia, adenopathy, mucocutaneous lesions, maculopapular rash with associated desquamation or bullae formation, condyloma lata, asymptomatic central nervous system invasion, and persistent rhinitis or “snuffles” [7, 12, 14]. Chorioretinitis, periostitis, or osteochondritis may also be seen. Late congenital syphilis presents in children over 2 years old. Signs may include Hutchinson′s teeth, Mulberry molars, interstitial keratitis, eighth cranial nerve deafness, hydrocephalus, frontal bossing, saddle nose deformity, saber shins, or seizures [7, 14].

### 3.2. Screening, Diagnosis, and Treatment

A crucial strategy in reducing the disease impact of syphilis is screening pregnant patients and sexually active individuals when indicated [14, 15]. Syphilis screening should occur at the initial prenatal visit for all pregnant patients, with follow‐up nontreponemal testing for those who test positive to monitor antibody titers. In settings with limited access to prenatal care, screening and treatment should occur as soon as pregnancy is identified [15]. Early detection is essential, as timely treatment of maternal syphilis in its initial stages can prevent congenital syphilis in nearly all cases [1, 4, 5]. Additional testing is recommended in the third trimester, in areas with high prevalence of congenital syphilis or after exposure to an infected partner, as well as at delivery for patients at higher risk [5, 15]. Treatment for pregnant individuals depends on the stage of infection, with a single intramuscular dose of penicillin being sufficient for primary, secondary, or early latent syphilis, whereas late latent or tertiary syphilis requires a three‐dose regimen [4, 5].

Diagnosis in a neonate with suspected congenital syphilis can be presumed with quantitative nontreponemal serologic titers more than fourfold of maternal titers at the time of delivery or positive *Treponema*‐specific immunoblot. However, definitive diagnosis requires the identification of spirochetes in samples of skin lesions or placenta, as previously mentioned [7, 12, 16]. Diagnosis may be aided by cerebrospinal fluid analysis for VDRL, complete blood count, platelet count, and long bone radiographs. Testing of an infant should also occur when a case of maternal syphilis is diagnosed in the first year postpartum. Infants born to mothers with known syphilis without a well‐documented and appropriate (penicillin based) treatment history, or in cases where infection occurred within 1 month prior to delivery, should be treated for presumed congenital syphilis [7]. Neonatal treatment involves a 10‐to‐14‐day course of penicillin G [12].

### 3.3. Multifactorial Health Impacts

Breakdowns in syphilis prevention during pregnancy remain a key contributor to the rise in congenital syphilis. Understanding why these prevention failures occur in practice, missed screenings and inadequate treatments in particular, is essential to inform effective strategies of action [1, 17, 18]. In 2022, congenital syphilis most often affected children of a birth parent that experienced at least one lapse in prevention efforts during pregnancy. This included roughly 37% with delayed or absent syphilis testing, 11% with a lack of documentation or no treatment, and 40% where treatment was insufficient [1]. In many cases, impediments to adequate treatment and testing do not stem from a single missed opportunity but rather from a combination of limited access, repeated disruptions, and ongoing barriers to prenatal care over time [4, 5]. Geographic factors and substance use are known factors that impact a patient′s ability to access ongoing, high‐quality prenatal care [5, 19]. Improving access to care and ensuring effective STI screening and treatment for adults may play an important role in preventing congenital syphilis and supporting better neonatal health in the United States.

Various public health interventions could be considered to decrease the rate of sexually transmitted and congenital infections in high‐risk communities. Examples include combining STI screening and other prenatal care services with substance use programs or making these services available at rehabilitation and treatment facilities. Additionally, increased outreach, campaigns, and community‐specific education focused on syphilis prevention, signs and symptoms to self‐monitor for, and the importance of prenatal care may help to target the most vulnerable populations.

In conclusion, the progressive increase in the incidence of syphilis infection within the United States is alarming. The rates of congenital syphilis are a major public health concern and will become a crisis if widespread transmission continues within the adult population.

## Funding

No funding was received for this manuscript.

## Ethics Statement

Informed consent was obtained from the patient′s mother for the publication of this case report. All identifying information has been removed to protect patient privacy. The authors affirm that all ethical guidelines were followed in accordance with institutional and publication standards.

## Conflicts of Interest

The authors declare no conflicts of interest.

## Data Availability

The data that support the findings of this study are available on request from the corresponding author. The data are not publicly available due to privacy or ethical restrictions.
